# Adverse Associations of Long-Term Exposure to PM_2.5_ and Its Components with Platelet Traits among Subway Shift-Workers without Air Purifier Use

**DOI:** 10.3390/toxics12080529

**Published:** 2024-07-23

**Authors:** Junling Liu, Pei Wang, Lv Shang, Fang Ye, Li Liu, Zhenyu He

**Affiliations:** 1 Wuhan Center for Disease Control and Prevention, Wuhan 430024, China; zhangyanjun@163.com (J.L.); pei.wang@whcdc.org (P.W.); 18627097901@163.com (L.S.); 2 School of Public Health, Tongji Medical College, Huazhong University of Science and Technology, Wuhan 430030, China; yefang@hust.edu.cn (F.Y.); liul2012@hust.edu.cn (L.L.)

**Keywords:** fine particulate matter, air purifier use, mean platelet volume, platelet distribution width, subway workers

## Abstract

Air purifier use, shift work, and long-term exposure to fine particulate matter (PM_2.5_) are linked to platelet abnormality. However, the role of air purifier use and shift work in the individual or joint associations of PM_2.5_ and its components with platelet indices are largely unknown. A total of 8772 participants were recruited from a population of subway workers in China. PM_2.5_ and its component data were obtained from the Tracking Air Pollution in China dataset. The role of air purifier use and shift work in the association between PM_2.5_ and its components and platelet indices were analyzed. Among shift workers without air purifier use, positive associations of PM_2.5_ and each component in PM_2.5_ with the mean platelet volume (MPV) or platelet counts (PLT) were observed, whereas negative associations of PM_2.5_ and each component in PM_2.5_ with the platelet distribution width (PDW) were observed. Furthermore, estimated changes (95%CIs) in PLT, MPV, and PDW in response to each 10th percentile increment in the mixture of PM_2.5_ and its components were 0.8657 (0.2496, 1.4819), 0.0192 (0.0054, 0.0329), and −0.0648 (−0.0945, −0.0351), respectively, and sulfate in PM_2.5_ was the major contributor to those associations. Long-term exposure to PM_2.5_ and its components was related to increased platelet disorders among shift workers without air purifier use, and those associations were mainly attributed to sulfate in PM_2.5_.

## 1. Introduction

Exposure to fine particulate matter (PM_2.5_) is linked to increased mortality from chronic diseases such as cardiovascular diseases and diabetes [1,2]. In recent years, researchers have paid attention to PM_2.5_ components associated with adverse human effects such as type 2 diabetes (T2D) and the aging process, as well as cardiovascular diseases [3,4,5]. Inflammatory and pro-thrombotic factors may be accepted as the potential mechanisms of adverse effects in relation to exposure to air pollution [6,7]. Limited studies have indicated that exposure to PM_2.5_ and its components are linked to platelet disorders [8,9]. Polycyclic aromatic hydrocarbons (PAHs), as one of the classical organic components bonded to PM_2.5_, are related to increased platelet activation [10,11], indicating that organically components in PM_2.5_ may play a vital role in PM_2.5_-exposure-related adverse human effects. Evidence suggests that inhaled PM_2.5_ can induce the activation of prothrombotic pathways by causing oxidative stress and inflammation, contributing to adverse vascular effects [12]. Furthermore, evidence indicates that exposure to organic extracts of particles can lead to oxidative stress of endothelial cells, which may be involved in cardiovascular disease [13,14]. As part of the above-mentioned evidence, platelet activation may be a potential biomarker of cardiovascular diseases. Thus, it is urgent to explore the association of PM_2.5_ and its components with platelet activation indices, which may shed new light on preventing cardiovascular disease.

Exposure to air pollution associated with platelet disorders has been well documented. The platelet indices, such as the mean platelet volume (MPV) or increase in platelet distribution width (PDW) and platelet counts (PLT), are easy to obtain and measure, which is useful for assessing platelet function [15]. Epidemiological studies show that exposure to PM_2.5_ is associated with MPV reduction or increased PDW and PLT [16,17,18]. Meanwhile, multiple-PAH metabolites are negatively associated with decreased ratios of MPV to PLT (MPVP), MPV, or PDW among preschool children [19]. However, two cross-sectional studies conducted in a middle-aged population show that multiple PAH metabolites are related to increased MPVP, MPV, and PDW [10,11]. Mechanism studies show that prombotic pathways induced by PM_2.5_ may be an important biological pathway involved in the development of cardiovascular risk [12,20]. For instance, toxicological studies have revealed that airborne PM can cause platelet aggregation and arterial thrombosis [21], which may result in the occurrence of CVD events. More attention should be paid to the association of the mixture of PM_2.5_ components with platelet indices to identify the major contributors, which may provide a potential mechanism for reducing the disease burden in association with PM_2.5_ component exposure.

Shift work has been recognized as an independent risk factor for human health. Over the past decade, accumulated evidence has demonstrated that shift work is associated with multiple adverse human effects, such as cardiovascular disorders and metabolic disorders, as well as breast cancer [22,23]. However, the potential underlying pathophysiological mechanisms are largely unclear. Several studies have suggested that low-grade inflammation may play a role in shift work-related diseases. For instance, one cross-sectional study conducted on airline employees showed that rotating shift work was related to the increased systemic inflammation C-reactive protein (CRP) and leukocyte count [24]. Nagai et al. reported that natural killer (NK) cell activity reduction was observed among night work nurses in comparison with day work nurses [25]. Furthermore, Nakao et al. conducted a study and reported that thromboxane (Tx) B2, as one important indicator of platelet activation, was significantly higher after the night shift than at baseline (median 65.3 vs. 180.4 ng/mL) [26]. In recent years, air purifier use in relation to health benefits has been gradually revealed. Liu et al. reported that air purifier use generated positive associations between long-term exposure to gaseous pollutants and systematic inflammation on smog days [27]. A meta-analysis also showed that PM_2.5_ exposure-related increases in inflammatory biomarkers such as CRP, interleukin 6 (IL-6), and tumor necrosis factor alpha (TNFα) were attenuated by the usage of portable air cleaners [28]. Platelet indices such as MPV have been recognized as inflammation biomarkers [29,30]. However, there is a lack of evidence on the potential modified effect of shift work and air purifier use on the associations of PM_2.5_ and its components with platelet indices.

Although few researchers have focused on the individual association of air purifier use and shift work or air pollution with inflammation, the modified effect of air purifier use and shift work on the associations of PM_2.5_ and components with platelet indices is largely unclear. Thus, in this study, we conducted a cross-sectional study among subway workers to assess the individual and joint associations of PM_2.5_ and its components with platelet indices (PLT, MPV, and PDW) and further identify whether these associations were modified by air purifier use or shift work. This study’s findings may identify the susceptible population and provide the underlying mechanism of air pollution-exposure-related adverse effects.

## 2. Material and Methods

### 2.1. Study Population

During the period from December 2018 to May 2019, a total of 11,960 metro workers were recruited from Wuhan Metro Group Co., Ltd., Wuhan, China. A questionnaire was used to collect individual information regarding individual characteristics (age, gender, body mass index (BMI)), socioeconomic status (education level, marital status, and annual family income), living environment conditions (such as housing type, housing surrounding environment, etc.), personal lifestyles (smoking and drinking statuses, sleep pattern, physical activity, etc.), as well as personal and family history of diseases. After excluding individuals with missing information on age, smoking and drinking statuses, educational levels, marital status, parameters of routine blood examination, as well as those who did not provide home addresses, a total of 8772 individuals were finally included for further analysis. This study was approved by the Wuhan Center for Disease Control and Prevention Ethics Committee (ethics approval No. 2018042). All individuals provided informed consent before carrying out this study.

### 2.2. Assessment of Shift Work and Air Purifier Use

Information regarding air purifier use was collected using the questionnaire. Individuals with self-reported usage of air purifiers were classified into air purifier use and non-using air purifier groups. Shift work was assessed by asking, “Does your work involve shift work?”. Shift work was defined as a “work schedule that falls outside of the normal daytime working hours”.

### 2.3. Estimated Concentrations of PM_2.5_ and Its Components

The concentrations of PM_2.5_ and its five components (sulfate (SO_4_^2−^), nitrate (NO_3_^−^), ammonium (NH_4_^+^), organic matter (OM), and black carbon (BC)) were obtained from the Tracking of Air Pollution in China database (http://tapdata.org.cn/, accessed on 10 July 2023). The estimated method was described elsewhere [31,32]. Briefly, the daily PM_2.5_ concentrations were estimated using a machine learning model with a 0.1° × 0.1° spatial resolution. The predictive performance of PM_2.5_ was assessed using out-of-bag cross-validation, and the result showed that R^2^ values for daily PM_2.5_ prediction were 83%. The PM_2.5_ components were derived from operational CMAQ simulations using PM_2.5_ components as constraints. To improve the accuracy of PM_2.5_ component measurements, the model was developed based on the observation data. The extreme gradient boosting algorithm was used to correct the relative contribution of PM_2.5_ component concentrations. The predicted PM_2.5_ components performed well with the surface measurements, and their R-values ranged from 0.67 to 0.80. We geocoded residential addresses for latitude and longitude for each individual using Google Maps and then extracted air pollutants from the nearest grid cell in which each residential address was located. The 3-year average PM_2.5_ and its components were used to reflect the long-term exposure to PM_2.5_ and its components, according to a previous study [3].

### 2.4. Platelet Parameters Measurements

All participants fasting for at least 8 h completed their health examination in the health examination center, which was assigned by the company of Wuhan Metro Group Co., Ltd., Wuhan, China. Venous blood sample of each individual was collected in ethylenediamine tetraacetic acid (EDTA) anticoagulation tubes by well-trained nurses. Subsequently, it was used for routine blood measurements on the same day. Platelet parameters such as PLT, MPV, and PDW were determined using a fully automated hematology analyzer.

### 2.5. Covariates’ Assessment

Educational levels were classified into <17 and ≥17 years groups. Marital status was classified into two groups: married/remarried/cohabitation and divorced/widowed/unmarried. Smoking status was classified as current smoker (every day or per week with at least seven cigarettes for at least six months), former smoker (not currently smoking cigarettes but had smoked at least seven cigarettes per week for at least six months in a lifetime), and never smoked (never smoked or fewer than 100 cigarettes smoked in their lifetime) groups [33]. Drinking status was classified as current drinker (some alcohol consumption in most weeks in the past year), former drinker (occasional or no alcohol consumption in the past year, but previously drank during most weeks), and never drank (no alcohol consumption in the past year and never drank during most weeks) groups. Individual physical activity was assessed using International Physical Activity Questionnaire. Briefly, the metabolic equivalents (METs) of physical activity were calculated based on the following formula: physical activity duration (hour/time) × frequency/week×metabolic equivalents (METs) coefficient of each type of activity and expressed METs (hours/week) [34]. Hypertension was defined by one of the following criteria: ① diastolic blood pressure (DBP) ≥ 90 mmHg or systolic blood pressure (SBP) ≥ 140 mmHg; ② self-reported hypertension diagnosed by a physician; ③ taking antihypertensive drugs. T2D was defined by one of the following criteria: ① self-reported hypertension diagnosed by a physician; ② fasting blood glucose value ≥ 7.0 mmol/L; ③ taking antidiabetic therapy. Dyslipidemia was defined by one of the following criteria: ① presence of one or more abnormal total cholesterol (TC), low-density lipoprotein cholesterol (LDL-C), high-density lipoprotein cholesterol (HDL-C), and triglyceride (TG) levels, which was defined by their corresponding levels being greater than 6.22 mmol/L, 2.26 mmol/L, 1.04 mmol/L, and 4.14 mmol/L; ② self-reported use of anti-dyslipidemia drugs in the past two-week.

### 2.6. Statistical Analysis

The categorical variables, such as gender and smoking and drinking statuses, were expressed as numbers with percentages, and the Chi-square test between usage and non-usage of air purifier groups was used to examine their distributions. The normal distribution of continuous variables such as age was displayed as mean with standard deviation (SD) and was compared using Student’s *t*-test. The non-normal distribution of continuous variables, such as platelet indices (MPV, PDW, and PLT) and their corresponding median differences, were compared using the Mann–Whitney U test. Three models were developed to assess the association of PM_2.5_ and its components with platelet indices (MPV, PDW, and PLT) by using generalized linear models: Model 1 was adjusted for age and gender. Model 2 was further adjusted for socioeconomic status (education levels, total family income, and marital status), lifestyles (smoking and drinking statuses and physical activity), and BMI. Model 3 added personal histories of hypertension, dyslipidemia, and type 2 diabetes.

Given that the traditional statistical analysis may be limited to identifying uncertain relationships between multiple components in mixtures, the Weighted Quantile Sum (WQS) regression model is widely used to assess the effects of exposure to all chemical mixtures [35], in which all pollutants were treated as a weighted additive index. Herein, a cumulative linear index was calculated by grouping each component of PM_2.5_ into quartiles, and the corresponding weight of each component of PM_2.5_ was used to reflect how it contributed to the whole WQS index. PM_2.5_ and its components’ exposures in relation to outcomes contained in the index were assumed in the same direction, which means that only positive or negative mixture effects on outcomes were assessed in the model. The estimated coefficient (β) was interpreted as the change in platelet indices in response to each quartile increment in the WQS index value after adjusting for Model 3 covariates. To further explore the potential modification factors, stratified analysis was used to identify the modification effect of air purifier use, shift work, or combination of them on associations of PM_2.5_ or its components with platelet indices. All data analysis were performed in R software version 4.3.0 (R Project for Statistical Computing). The statistical significances were set with two-sided *p* values of less than 0.05.

## *3.* Results

### 3.1. Characteristics of the Study Population

Table 1 shows that the average age of individuals who did not use air purifiers was lower than that of individuals who used air purifiers (26.9 vs. 28.8 years). The mean MET value of physical activity in individuals who did not use air purifiers was lower than that in individuals who used air purifiers (10.1 vs. 8.9 h/week). Meanwhile, the mean BMI in individuals who did not use air purifiers was lower than that of individuals who used air purifiers (23.8 vs. 23.2 kg/m^2^). Except for smoking and drinking statuses as well as personal histories of T2D and hypertension, differences in the distributions of the other selected variables were found between usage and non-usage of air purifier groups (all *p* < 0.05). The mean level of PDW was found to be different between groups who used and did not use air purifiers (*p* < 0.05).

Table 2 shows the distributions and correlations of PM_2.5_ and its components. The results showed that the median PM_2.5_, SO_4_^2−^, NO_3_^−^, NH_4_^+^, OM, or BC was 53.61, 9.63, 11.58, 6.93, 13.51, or 2.62. A moderate–high correlation between each paired PM_2.5_ and its components was found (r ranged from 0.559 to 0.994).

### 3.2. Individual Associations of PM_2.5_ and Its Components with Platelet Parameters

As shown in Table 3, Model 1 shows that the estimated β (95%CI) of PLT in response to each natural-log transformed increment in PM_2.5_, SO_4_^2−^, NO_3_^−^, NH_4_^+^, OM, and BC was 13.6 (3.6, 23.6), 14.2 (4.6, 23.8), 8.8 (−1.9, 19.5), 7.2 (−3.0, 17.4), 11.2 (2.3, 20.1), and 10.9 (2.4, 19.4), respectively. The corresponding β (95%CI) of MPV were 0.4 (0.1, 0.6), 0.5 (0.3, 0.7), 0.3 (0.1, 0.6), 0.3 (0.1, 0.5), 0.3 (0.1, 0.5), and 0.3 (0.1, 0.5); of PDW were −1.2 (−1.7, −0.7), −1.3 (−1.8, −0.9), −1.0 (−1.5, −0.5), −0.9 (−1.3, −0.4), −0.9 (−1.4, −0.5), and −0.9 (−1.3, −0.5). Models 2–3 show that the results were not a substantial change from Model 1.

Table 4 displays the difference in associations of PM_2.5_ and its components with platelet parameters (PLT, MPV, and PDW) between individuals who did and did not use air purifiers after adjusting for potential confounders. The results showed that in response to each natural-log transformed increment in PM_2.5_, SO_4_^2−^, NO_3_^−^, NH_4_^+^, OM, and BC, the estimated β (95%CI) of PLT was 16.7 (6.6, 26.7), 16.5 (6.9, 26.1), 10.6 (−0.2, 21.3), 8.8 (−1.5, 19.1), 14.1 (5.2, 23.1), and 13.7 (5.1, 22.3), respectively. The matched corresponding β (95%CI) of MPV were 0.3 (0.1, 0.5), 0.4 (0.2, 0.6), 0.3 (0.0, 0.5), 0.2 (0.0, 0.5), 0.2 (0.0, 0.4), and 0.2 (0.0, 0.4); those of PDW were −1.2 (−1.7, −0.7), −1.3 (−1.8, −0.9), −1.0 (−1.5, −0.5), −0.8 (−1.3, −0.3), −1.0 (−1.4, −0.5), and −0.9 (−1.4, −0.5). However, the associations mentioned above were not found among individuals who used air purifiers.

As shown in Table 5, differences in the associations of PM_2.5_ and its components with platelet parameters (PLT, MPV, and PDW) were explored between shift and non-shift workers. In response to each natural-log transformed increment in PM_2.5_, SO_4_^2−^, NO_3_^−^, NH_4_^+^, OM, and BC, the estimated βs (95%CIs) of PLT were 20.9 (8.7, 33.1), 21.4 (9.6, 33.2), 16.4 (3.3, 29.6), 14.4 (1.9, 26.9), 17.1 (6.2, 28.0), and 16.1 (5.7, 26.5), respectively. The matched corresponding βs (95%CIs) of MPV were 0.39 (0.11, 0.66), 0.50 (0.24, 0.76), 0.30 (0.01, 0.59), 0.26 (−0.01, 0.54), 0.27 (0.03, 0.51), and 0.28 (0.05, 0.51); those of PDW were −1.37 (−1.95, −0.79), −1.52 (−2.08, −0.96), −1.14 (−1.76, −0.51), −0.99 (−1.58, −0.39), −1.05 (−1.57, −0.54), and −1.02 (−1.52, −0.53). However, the associations mentioned above were not found among non-shift workers.

### 3.3. The Mixture of PM_2.5_ and Its Components with Platelet Parameters

As shown in Table 6, the results showed that the estimated βs (95%CIs) of PLT, MPV, and PDW in response to each 10th percentile increment in the mixture of PM_2.5_ and its components was 0.6925 (0.196, 1.1889), 0.0124 (0.0014, 0.0233), or −0.0568 (−0.0802, −0.0333), after adjusting for age and gender; socioeconomic status (education levels, total family income, and marital status); lifestyles (smoking and drinking statuses and physical activity); BMI; and air purifier use; as well as personal histories of hypertension, dyslipidemia, and type 2 diabetes. The results from the stratified analysis by the usage and non-usage of air purifiers showed that associations of the mixture of PM_2.5_ and its components with platelet parameters were only observed among individuals who did not use air purifiers after being adjusted for potential confounders. The results showed that the estimated β (95%CI) of PLT, MPV, and PDW in response to each 10th percentile increment in the mixture of PM_2.5_ and its components was 0.7961 (0.2911, 1.3011), 0.0173 (0.006, 0.0287), and −0.0551 (−0.0842, −0.026) after adjusting for age and gender; socioeconomic status (education levels, total family income, and marital status); lifestyles (smoking and drinking statuses and physical activity); BMI; and shift work status; as well as personal histories of hypertension, dyslipidemia, and type 2 diabetes. The results from the stratified analysis by shift work status showed that associations of the mixture of PM_2.5_ and its components with platelet parameters were only observed among individuals with shift work after adjusting for potential confounders.

### 3.4. The Individual and Mixture of PM_2.5_ and Its Components with Platelet Parameters by Air Purifier and Shift Work

**Figure 1:**
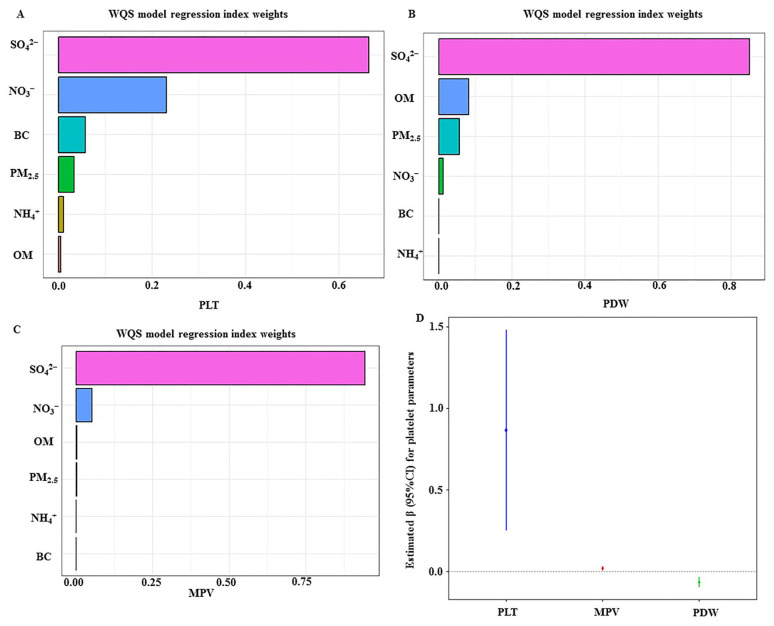
WQS model regression index weights for the PLT (**A**), PDW (**B**), and MPV (**C**); also shown are the estimated changes and their corresponding 95%CIs for PLT, PDW, and MPV (**D**) among shift workers without air purifier use. The model was adjusted for age and gender; socioeconomic status (education levels, total family income, and marital status); lifestyles (smoking and drinking statuses and physical activity); BMI; and shift work; as well as personal histories of hypertension, dyslipidemia, and type 2 diabetes. The dotted line in (**D**) represents the reference line of the estimated βs (95%CIs).

As shown in Table 7, associations of PM_2.5_ and its components with platelet indices were investigated. The results showed that in response to each natural-log transformed increment in PM_2.5_, SO_4_^2−^, NO_3_^−^, NH_4_^+^, OM, and BC, the estimated βs (95%CIs) of PLT were 21.4 (8.2, 34.5), 21.5 (8.8, 34.2), 15.2 (1.1, 29.3), 13.6 (0.1, 27.0), 17.7 (6.0, 29.4), and 16.7 (5.6, 27.9), respectively. The corresponding βs (95%CIs) of MPV were 0.42 (0.13, 0.72), 0.56 (0.28, 0.85), 0.38 (0.06, 0.70), 0.33 (0.03, 0.64), 0.29 (0.02, 0.55), and 0.29 (0.04, 0.54); those of PDW were −1.47 (−2.10, −0.84), −1.65 (−2.26, −1.04), −1.21 (−1.88, −0.53), −1.05 (−1.69, −0.40), −1.12 (−1.68, −0.56), and −1.09 (−1.62, −0.55) among shift workers without air purifier use. No associations of PM_2.5_ and its components with platelet indices were found among non-shift workers with air purifier use, non-shift workers without air purifier use, and shift workers with air purifier use. Furthermore, we explored associations of the mixture of PM_2.5_ and its components with platelet indices using the WQS model. Figure 1 showed that the SO_4_^2−^ was the highest-weighted PM_2.5_ and its components in PLT, PDW and MPV. Meanwhile, the estimated changes (95%CI) in PLT, MPV, and PDW in response to each 10th-percentile increment in the mixture of PM_2.5_ and its components were 0.8657 (0.2496, 1.4819), 0.0192 (0.0054, 0.0329), and −0.0648 (−0.0945, −0.0351), respectively, after adjusting for potential confounders. A sensitive analysis was further conducted to explore associations between the mixture of PM_2.5_ and its components with platelet parameters among never-smoking shift workers without air purifier use, and the results did not indicate substantial changes (Supplementary Table S1).

## 4. Discussions

The findings of this study showed that long-term exposure to PM_2.5_ was related to increased platelet count, which was in line with previous studies. The Heinz Nixdorf Recall Study showed that each 2.4 µg/m^3^ increment in PM_2.5_ level was associated with a 2.3% (95%CI: 1.4%, 3.3%) increased PLT count in 4814 German adults [17]. A large cohort study showed that each 10 µg/m^3^ increment in the 2-year average of PM_2.5_ concentration was associated with a 0.42% (95%CI: 0.38%, 0.47%) or 0.49% (95%CI: 0.44%, 0.54%) increased PLT count for males or females in Taiwan [18]. A cross-sectional study conducted in three Chinese cities indicated that each 10 μg/m^3^ increment in PM_2.5_ concentration was related to a 1.04% (95%CI: 0.16%, 1.92%) increased PLT among middle-aged individuals [36]. However, evidence also suggested that exposure to PM_2.5_ was related to decreased PLT values. For instance, a panel study showed that exposure to PM_2.5_ was related to decrement in PLT count among obese young adults [37]. A large cross-sectional study conducted in a Chinese rural population showed that exposure to PM_2.5_ was associated with a decrement in PLT count [9]. Furthermore, exposure to the mixture of PM_2.5_ and its components related to increased PLT counts may indirectly support the findings of previous studies. For instance, Zhu et al. reported that elevated heavy metal components in PM_2.5_ were associated with increased PLT count in male apoE^−/−^ mice [38]. As one classical component of PM_2.5_, exposure to PAHs was related to increased PLT counts. However, Wang et al. reported that water-soluble ions in PM_2.5_ were related to a decrease in PLT counts [8].

Both MPV and PDW have been used as early indexes of platelet activation. This study’s finding suggested that exposure to PM_2.5_ and its components was related to an increment in MPV value but associated with a decrement in PDW value. However, evidence showed that exposure to PM_2.5_ was related to increased MPV and PDW [9]. Associations of exposure to PAHs with MPV were not inconsistent. One cross-sectional study indicated that PAH exposure was related to increased MPV in a general Chinese population (*n* = 3847) [10]. However, another study showed that exposure to PAHs was related to decreased MPV and PDW among children aged 2–7 (*n* = 239) [19]. Furthermore, PM-bound metals-related platelet dysfunction has also been reported [39]. The difference in results between this study and the previous study may be partly explained by the differences in study design, the level and components of PM_2.5_, and the sample size of the study population. PDW can indicate platelet heterogeneity and variation in platelet size. Fu et al. reported that decreased MPV and increased PDW were found among laryngeal cancer patients compared to control subjects [40]. This study’s findings suggested that exposure to PM_2.5_ and its components related to decreased PDW and increased MPV may be plausible; due to this, increased MPV may reduce platelet heterogeneity.

This study indicated that exposure to PM_2.5_ and its components was related to alterations of platelet indexes among individuals who did not use air purifiers but not in individuals who used air purifiers. Several studies have shown that air purifier use may reduce PM_2.5_ exposure to decrease concentrations of inflammatory biomarkers [28]. Dubey et al. reported that air purifier use could reduce different sizes of PM concentrations by 12–73% [41]. Liu et al. found that using an air purifier is cost-effective in reducing PM_2.5_ exposure-related deaths by achieving indoor PM_2.5_ concentrations of 35 or 25 μg/m^3^ [42]. Moreover, Hansel et al. conducted a randomized clinical trial that indicated that portable high-efficiency particulate absolute air cleaners had health benefits on indoor air pollution related to respiratory morbidity among former smokers with chronic obstructive pulmonary disease [43]. The evidence mentioned above suggests that air purifier use may reduce air pollution-related adverse effects by leading to a decrement in air pollution levels.

Shift workers, as a vulnerable population, may suffer from compromised sleep length, poor sleep quality, and increased risks of chronic diseases such as hypertension and diabetes, even after retirement for years [44]. In recent years, accumulated evidence has shown that platelet functions may interrelate and activate an inflammation response, further involved in the development of multiple chronic diseases [45]. A noteworthy finding of this study is that the increase in PM_2.5_ or each of its components was associated with increased platelet activation among subway shift work workers who needed to pay more attention. The result may be indirectly supported by several previous studies. Velazquez-Kronen et al. conducted a study among those aged ≥45 years in the US population, and they reported that shift workers had higher inflammatory markers such as CRP and white blood cells in comparison with day workers [46]. El-Benhawy et al. reported that serum inflammatory markers among night shift workers were higher than those of the day shift control group of workers [47]. A pilot study showed that shift workers may have high levels of low-grade systemic inflammation indicators (IL-10 and TNF-α) compared to those in day workers [48]. Furthermore, Nakao et al. conducted an observational study, and they revealed that thromboxane (Tx) B2, which is one of the most important markers of platelet activation, was significantly higher after the night shift than at baseline (median 65.3 vs. 180.4 ng/mL) [26]. Taken together with the evidence mentioned above, we may infer that exposure to PM_2.5_ and its components with increased platelet activation among subway shift workers who do not use air purifiers may be due to increased air pollutant exposure and population susceptibility to air pollution-exposure-related adverse effects.

Another notable finding was that SO_4_^2−^ of PM_2.5_ plays a major role in platelet dysfunction among subway shift workers who do not use air purifiers. Particulate SO_4_^2−^ is mainly produced in complex reactions to gas emissions from fossil fuel combustion, industrial activities (such as steel production), and agriculture [49], and acidic particles are associated with some of the most adverse human health consequences [50]. Both population studies found that exposure to SO_4_^2−^ affected levels of IL-1β, IL-5, IL-7, IL-12, and IFN-γ, increasing the risk of metabolic syndrome, suggesting that SO_4_^2−^ affects systemic inflammation and metabolic disease [51,52]. Understandably, workers are exposed to high concentrations of pollutants due to the lack of air circulation in the working environment, and the unhealthy lifestyle of shift workers makes them more susceptible to health damage from pollutants due to low body resistance. Thus, measures to reduce these components of environmental pollutants, such as low-sulfur treatment of fuels, regular air monitoring, and enhanced air circulation in the workplace, may have greater public health benefits.

The mechanistic research showed that air pollution-induced activation of platelet inflammation occurred via the induction of oxidative stress in the human body. For instance, PM- or PM-bounded chemical components such as PAHs and metals can lead to oxidative stress, promote a pro-inflammatory response, and ultimately initiate platelet activation [12]. Exposure to air pollution may stimulate megakaryocytes to generate new platelets and consume older platelets [53,54]. Evidence has demonstrated that larger or younger platelets might cause prominent adverse effects such as inflammation response and atherosclerosis by elevating the secretion of inflammatory cytokines (such as IL-6 and CRP) compared to older platelets [55]. Not only that, in the context of air pollution, early molecular events such as cellular DNA and chromosome damage may have an impact on the onset and development of non-communicable diseases, including cancer. Specifically, this involves blood cells acting as replacement cells for whole-body exposure [56]. As per the evidence mentioned above, we may infer that this study’s findings were reasonable.

Several limitations should be mentioned: first, because of the nature of the cross-sectional study, the causal associations of exposure to PM_2.5_ and its components with platelets function could not be inferred. Second, the recall bias could not be avoided because individuals’ information, such as lifestyle behaviors and socioeconomic status, was obtained using a questionnaire. Third, misclassification exposure in this study may exist because individuals’ air pollution exposure assessment occurred by geocoding each individual’s address. Fourth, other platelet function tests, such as aggregation, were not evaluated. Fifth, we will measure the serum cotinine levels to assess smoking exposure for each participant based on previous studies’ findings, which suggested that serum cotinine is widely used to classify smoking status [57]. Sixth, although we controlled several important confounders, other unmeasured or unselected variables (such as indoor air pollution levels) were not measured. Last but not least, considering that accumulated evidence has revealed the effects of the pandemic on the air pollution levels change and its related adverse human health [58,59,60], we will explore the effect of the pandemic on the associations of air pollution and platelet parameters in the ongoing study carried out among subway workers.

## 5. Conclusions

Long-term exposure to PM_2.5_ and its components was related to increased platelet counts and MPV but decreased PDW. Those associations were only found among subway shift workers who did not use air purifiers, implying that sulfate is the priority control pollutant of components in PM_2.5_ and air purifier use may be an effective intervention to prevent PM_2.5_ exposure-related platelet dysfunction involved in the development of disease among subway shift workers.

## Figures and Tables

**Table 1:** Distributions of selected variables of the participants.

Variables	Total Population (*n* = 8772)	Air Purifier Use		*p*-Values	Shift-Work		*p*-Values
		No	Yes		No	Yes	
Age (year, mean ± SD)	27.2 ± 4.1	26.9 ± 4.0	28.8 ± 4.4	<0.001 ^a^	28.4 ± 5.2	26.6 ± 3.4	<0.001 ^a^
Gender (*n*, %)				<0.001 ^b^			<0.001 ^b^
Males	7024 (80.1)	6066 (81.2)	958 (73.4)		2413 (89.5)	4602 (75.9)	
Females	1748 (19.9)	1400 (18.8)	348 (26.6)		284 (10.5)	1462 (24.1)	
Education level (*n*, %)				0.029 ^b^			<0.001 ^b^
<17 years	8615 (98.2)	7342 (98.3)	1273 (97.5)		2549 (94.5)	6055 (99.9)	
≥17 years	157 (1.8)	124 (1.7)	33 (2.5)		148 (5.5)	9 (0.1)	
Marital status (*n*, %)				<0.001 ^b^			<0.001 ^b^
Married/living together	3148 (35.9)	2320 (31.1)	828 (63.4)		1145 (42.5)	1997 (32.9)	
Divorced/widowed/separated/Unmarried	5624 (64.1)	5146 (68.9)	478 (36.6)		1552 (57.5)	4067 (67.1)	
Total family income (n, %)				<0.001 ^b^			<0.001 ^b^
<100,000 RMB	3740 (44.0)	3354 (46.3)	386 (30.6)		1136 (43.6)	2599 (44.1)	
100,000 RMB	2479 (29.1)	2119 (29.2)	360 (28.6)		728 (27.9)	1749 (29.7)	
>150,000 RMB	2288 (26.9)	1774 (24.5)	514 (40.8)		743 (28.5)	1541 (26.2)	
Smoking status (*n*, %)				0.129 ^b^			0.003 ^b^
Never	6559 (74.8)	5603 (75)	956 (73.2)		1961 (72.7)	4591 (75.7)	
Former	278 (3.2)	226 (3.0)	52 (4.0)		104 (3.9)	174 (2.9)	
Current	1935 (22.1)	1637 (21.9)	298 (22.8)		632 (23.4)	1299 (21.4)	
Drinking status (*n*, %)				0.566 ^b^			<0.001 ^b^
Never	7010 (79.9)	5969 (79.9)	1041 (79.7)		2056 (76.2)	4946 (81.6)	
Former	175 (2.0)	144 (1.9)	31 (2.4)		74 (2.7)	100 (1.6)	
Current	1587 (18.1)	1353 (18.1)	234 (17.9)		567 (21.0)	1018 (16.8)	
Passive smoking (*n*, %)				0.027 ^b^			<0.001 ^b^
Yes	4652 (54.1)	3998 (54.6)	654 (51.3)		1540 (58.2)	3106 (52.3)	
No	3940 (45.9)	3319 (45.4)	621 (48.7)		1107 (41.8)	2828 (47.7)	
Physical activity-MET (h/week, mean ± SD)	9.1 ± 17.7	10.1 ± 19.0	8.9 ± 17.5	0.004 ^c^	10.2 ± 17.9	8.5 ± 17.6	<0.001 ^c^
Body mass index (kg/m^2^, mean ± SD)	23.3 ± 3.7	23.8 ± 3.8	23.2 ± 3.6	<0.001 ^a^	23.7 ± 3.6	23.1 ± 3.7	<0.001 ^a^
Dyslipidemia (*n*, %)				<0.001 ^b^			<0.001 ^b^
Yes	2542 (29.0)	2106 (28.2)	436 (33.4)		873 (32.4)	1666 (27.5)	
No	6228 (71.0)	5359 (71.8)	869 (66.6)		1824 (67.6)	4396 (72.5)	
T2D (*n*, %)				0.841 ^b^			0.020 ^b^
Yes	85 (1.0)	73 (1.0)	12 (0.9)		36 (1.3)	49 (0.8)	
No	8687 (99.0)	7393 (99.0)	1294 (99.1)		2661 (98.7)	6015 (99.2)	
Hypertension (*n*, %)				0.247 ^b^			0.139 ^b^
Yes	912 (10.4)	788 (10.6)	124 (9.5)		299 (11.1)	609 (10.0)	
No	7860 (89.6)	6678 (89.4)	1182 (90.5)		2398 (88.9)	5455 (90.0)	
Platelet parameters							
PLT (10^9^/L, median, IQR)	212 (182, 245)	212 (181, 244)	213 (184, 246)	0.214 ^c^	211 (180, 244)	212 (183, 245)	0.053 ^c^
MPV (fL, median, IQR)	9.2 (8.4, 10)	9.2 (8.4, 10.0)	9.3 (8.5, 10.0)	0.454 ^c^	9.4 (8.5, 10.1)	9.2 (8.4, 9.9)	<0.001 ^b^
PDW (fL, median, IQR)	15.4 (11.7, 15.9)	15.4 (11.7, 15.9)	15.3 (11.5, 15.9)	0.015 ^c^	15.2 (11.5, 15.9)	15.4 (11.7, 15.9)	0.009 ^c^

SD: standard deviation; T2D: type 2 diabetes; PLT: platelet counts; MPV: mean platelet volume; PDW: platelet distribution width. ^a^ Student’s *t* test was used to compare normally distributed continuous variables between groups who did and did not use air purifiers; ^b^ A Chi-square test was used to test the distributions of categorical variables between groups who did and did not use air purifiers; ^c^ Mann–Whitney U test was used to test the distributions of categorical variables between groups who did and did not use air purifiers.

**Table 2:** Three-year average concentrations and pairwise correlations of PM_2.5_ and its components.

Variables	3-Year Average Concentrations					Spearman Correlation Coefficients					
	Mean	Median	Minimum	Maximum	IQR	PM_2.5_	SO_4_^2−^	NO_3_^−^	NH_4_^+^	OM	BC
PM_2.5_ (μg/m^3^)	56.24	53.61	46.44	75.65	9.77	1.00					
SO_4_^2−^ (μg/m^3^)	9.61	9.63	8.25	14.86	1.35	0.933 **	1.00				
NO_3_^−^ (μg/m^3^)	11.77	11.58	10.41	18.98	1.76	0.798 **	0.917 **	1.00			
NH_4_^+^ (μg/m^3^)	7.09	6.93	6.22	12.44	1.10	0.788 **	0.906 **	0.990 **	1.00		
OM (μg/m^3^)	14.30	13.51	11.10	18.73	2.30	0.939 **	0.790 **	0.608 **	0.588 **	1.00	
BC (μg/m^3^)	2.77	2.62	2.08	3.71	0.50	0.929 **	0.771 **	0.579 **	0.559 **	0.994 **	1.00

** *p* < 0.01; IQR: interquartile range; PM_2.5_: fine particulate matter; sulfate: SO_4_^2−^; nitrate: NO_3_^−^; ammonium: NH_4_^+^; BC: black carbon; organic matter: OM.

**Table 3:** Associations of exposure to PM_2.5_ and its components with platelet parameters.

Variables	Model 1 (β, 95%CI)	Model 2 (β, 95%CI)	Model 3 (β, 95%CI)
PLT			
PM_2.5_	13.6 (3.6, 23.6)	17.2 (7.1, 27.2)	16.7 (6.7, 26.8)
SO_4_^2−^	14.2 (4.6, 23.8)	17.2 (7.5, 26.8)	16.5 (6.9, 26.2)
NO_3_^−^	8.8 (−1.9, 19.5)	10.9 (0.1, 21.7)	10.6 (−0.2, 21.4)
NH_4_^+^	7.2 (−3.0, 17.4)	9.2 (−1.1, 19.4)	8.8 (−1.4, 19.1)
OM	11.2 (2.3, 20.1)	14.5 (5.5, 23.5)	14.2 (5.2, 23.2)
BC	10.9 (2.4, 19.4)	14.1 (5.5, 22.7)	13.7 (5.1, 22.3)
MPV			
PM_2.5_	0.4 (0.1, 0.6)	0.3 (0.1, 0.5)	0.3 (0.1, 0.5)
SO_4_^2−^	0.5 (0.3, 0.7)	0.4 (0.2, 0.6)	0.4 (0.2, 0.6)
NO_3_^−^	0.3 (0.1, 0.6)	0.3 (0.0, 0.5)	0.3 (0.0, 0.5)
NH_4_^+^	0.3 (0.1, 0.5)	0.2 (0.0, 0.5)	0.2 (0.0, 0.5)
OM	0.3 (0.1, 0.5)	0.2 (0.0, 0.4)	0.2 (0.0, 0.4)
BC	0.3 (0.1, 0.5)	0.2 (0.0, 0.4)	0.2 (0.0, 0.4)
PDW			
PM_2.5_	−1.2 (−1.7, −0.7)	−1.2 (−1.7, −0.7)	−1.2 (−1.7, −0.7)
SO_4_^2−^	−1.3 (−1.8, −0.9)	−1.3 (−1.8, −0.9)	−1.3 (−1.8, −0.9)
NO_3_^−^	−1.0 (−1.5, −0.5)	−1.0 (−1.5, −0.4)	−1.0 (−1.5, −0.4)
NH_4_^+^	−0.9 (−1.3, −0.4)	−0.8 (−1.3, −0.3)	−0.8 (−1.3, −0.3)
OM	−0.9 (−1.4, −0.5)	−1.0 (−1.4, −0.5)	−1.0 (−1.4, −0.5)
BC	−0.9 (−1.3, −0.5)	−0.9 (−1.4, −0.5)	−0.9 (−1.4, −0.5)

PLT: platelet counts; PM_2.5_: fine particulate matter; SO_4_^2−^: sulfate; NO_3_^−^: nitrate; NH^4+^: ammonium; BC: black carbon; OM: organic matter; MPV: mean platelet volume; PDW: platelet distribution width. Model 1 was adjusted for age and gender; Model 2 was further adjusted for socioeconomic status (education levels, total family income, and marital status), lifestyles (smoking and drinking statuses and physical activity), and BMI; Model 3 added personal histories of hypertension, dyslipidemia, and type 2 diabetes.

**Table 4:** Associations of exposure to PM_2.5_ and its components with platelet parameters: stratified analysis by usage and non-usage of air purifiers.

Variables	PLT ^a^ (β, 95%CI)	MPV ^a^ (β, 95%CI)	PDW ^a^ (β, 95%CI)
Usage of air purifiers			
PM_2.5_	9.4 (−16.6, 35.3)	0.0 (−0.6, 0.6)	−0.4 (−1.7, 0.8)
SO_4_^2−^	13.7 (−11.5, 38.8)	0.1 (−0.5, 0.6)	−0.5 (−1.8, 0.7)
NO_3_^−^	18.2 (−10.6, 47.1)	−0.2 (−0.8, 0.5)	−0.7 (−2.1, 0.6)
NH_4_^+^	14.4 (−13.2, 42.1)	−0.1 (−0.7, 0.5)	−0.7 (−2.0, 0.7)
OM	5.3 (−18.0, 28.6)	0.0 (−0.5, 0.5)	−0.3 (−1.4, 0.8)
BC	4.6 (−17.7, 27.0)	0.0 (−0.5, 0.5)	−0.2 (−1.3, 0.8)
Non-usage of air purifiers			
PM_2.5_	16.7 (6.6, 26.7)	0.3 (0.1, 0.5)	−1.2 (−1.7, −0.7)
SO_4_^2−^	16.5 (6.9, 26.1)	0.4 (0.2, 0.6)	−1.3 (−1.8, −0.9)
NO_3_^−^	10.6 (−0.2, 21.3)	0.3 (0.0, 0.5)	−1.0 (−1.5, −0.4)
NH_4_^+^	8.8 (−1.5, 19.1)	0.2 (0.0, 0.5)	−0.8 (−1.3, −0.3)
OM	14.1 (5.2, 23.1)	0.2 (0.0, 0.4)	−1.0 (−1.4, −0.5)
BC	13.7 (5.1, 22.3)	0.2 (0.0, 0.4)	−0.9 (−1.4, −0.5)

PLT: platelet counts; PM_2.5_: fine particulate matter; SO_4_^2−^: sulfate; NO_3_^−^: nitrate; NH_4_^+^: ammonium; BC: black carbon; OM: organic matter; MPV: mean platelet volume; PDW: platelet distribution width. ^a^ Adjusted for age and gender; socioeconomic status (education levels, total family income, and marital status); lifestyles (smoking and drinking statuses and physical activity); and BMI; as well as personal histories of hypertension, dyslipidemia, and type 2 diabetes.

**Table 5:** Associations of exposure to PM_2.5_ and its components with platelet parameters: stratified analysis by shift work.

Variables	PLT ^a^ (β, 95%CI)	MPV ^a^ (β, 95%CI)	PDW ^a^ (β, 95%CI)
Shift work (Yes)			
PM_2.5_	20.9 (8.7, 33.1)	0.39 (0.11, 0.66)	−1.37 (−1.95, −0.79)
SO_4_^2−^	21.4 (9.6, 33.2)	0.50 (0.24, 0.76)	−1.52 (−2.08, −0.96)
NO_3_^−^	16.4 (3.3, 29.6)	0.30 (0.01, 0.59)	−1.14 (−1.76, −0.51)
NH_4_^+^	14.4 (1.9, 26.9)	0.26 (−0.01, 0.54)	−0.99 (−1.58, −0.39)
OM	17.1 (6.2, 28.0)	0.27 (0.03, 0.51)	−1.05 (−1.57, −0.54)
BC	16.1 (5.7, 26.5)	0.28 (0.05, 0.51)	−1.02 (−1.52, −0.53)
Shift work (No)			
PM_2.5_	7.3 (−10.4, 24.9)	0.04 (−0.38, 0.47)	−0.73 (−1.61, 0.15)
SO_4_^2−^	6.7 (−10.1, 23.5)	0.20 (−0.2, 0.61)	−0.76 (−1.6, 0.07)
NO_3_^−^	−0.7 (−19.5, 18)	0.10 (−0.36, 0.55)	−0.45 (−1.38, 0.48)
NH_4_^+^	−2 (−19.9, 15.8)	0.08 (−0.35, 0.51)	−0.35 (−1.24, 0.54)
OM	6.8 (−9.2, 22.7)	−0.04 (−0.42, 0.35)	−0.58 (−1.37, 0.21)
BC	7.5 (−7.8, 22.8)	−0.03 (−0.4, 0.34)	−0.59 (−1.35, 0.17)

PLT: platelet counts; PM_2.5_: fine particulate matter; SO_4_^2−^: sulfate; NO_3_^−^: nitrate; NH_4_^+^: ammonium; BC: black carbon; OM: organic matter; MPV: mean platelet volume; PDW: platelet distribution width. ^a^ Adjusted for age and gender; socioeconomic status (education levels, total family income, and marital status); lifestyles (smoking and drinking statuses and physical activity); and BMI; as well as personal histories of hypertension, dyslipidemia, and type 2 diabetes.

**Table 6:** Associations of the mixture of PM_2.5_ and its components with platelet parameters.

Variables	PLT (β, 95%CI) ^a^	MPV (β, 95%CI) ^a^	PDW (β, 95%CI) ^a^
Total population ^a^	0.6925 (0.196, 1.1889)	0.0124 (0.0014, 0.0233)	−0.0568 (−0.0802, −0.0333)
Usage of air purifier ^b^	−0.376 (−1.6725, 0.9206)	0.0022 (−0.0249, 0.0292)	−0.0204 (−0.0812, 0.0404)
Non-usage of air purifier ^b^	0.7886 (0.2471, 1.3301)	0.0111 (−0.0005, 0.0227)	−0.0513 (−0.0762, −0.0264)
Total population^c^	0.7961 (0.2911, 1.3011)	0.0173 (0.006, 0.0287)	−0.0551 (−0.0842, −0.026)
Shift work (Yes)	1.0441 (0.4300, 1.6582)	0.0127 (−0.0004, 0.0258)	−0.0204 (−0.0812, 0.0404)
Shift work (No)	0.1133 (−0.7545, 0.9812)	0.0073 (−0.0137, 0.0284)	−0.0080 (−0.0516, 0.0357)

PLT: platelet counts; PM_2.5_: fine particulate matter; SO_4_^2−^: sulfate; NO_3_^−^: nitrate; NH_4_^+^: ammonium; BC: black carbon; OM: organic matter; MPV: mean platelet volume; PDW: platelet distribution width. ^a^ Adjusted for age and gender; socioeconomic status (education levels, total family income, and marital status); lifestyles (smoking and drinking statuses and physical activity); BMI; and air purifier use; as well as personal histories of hypertension, dyslipidemia, and type 2 diabetes. ^b^ Adjusted for age and gender; socioeconomic status (education levels, total family income, and marital status); lifestyles (smoking and drinking statuses and physical activity); and BMI; as well as personal histories of hypertension, dyslipidemia, and type 2 diabetes. c Adjusted for age and gender; socioeconomic status (education levels, total family income, and marital status); lifestyles (smoking and drinking statuses and physical activity); BMI; and shift work; as well as personal histories of hypertension, dyslipidemia, and type 2 diabetes.

**Table 7:** Associations of exposure to PM_2.5_ and its components with platelet parameters: stratified analysis by shift work and air purifier use.

Shift-Work	Air Purifier Use	PLT ^a^ (β, 95%CI)	MPV ^a^ (β, 95%CI)	PDW ^a^ (β, 95%CI)
Yes	Yes			
PM_2.5_		14.9 (−19.2, 49)	0.04 (−0.65, 0.73)	−0.44 (−1.98, 1.10)
SO_4_^2−^		18 (−14.8, 50.8)	0.01 (−0.65, 0.68)	−0.45 (−1.93, 1.03)
NO_3_^−^		21.3 (−15.6, 58.1)	−0.30 (−1.05, 0.45)	−0.41 (−2.07, 1.26)
NH_4_^+^		16.9 (−18.3, 52.1)	−0.27 (−0.98, 0.44)	−0.33 (−1.93, 1.26)
OM		10.4 (−20.2, 41)	0.05 (−0.57, 0.67)	−0.36 (−1.75, 1.02)
BC		9.5 (−19.8, 38.8)	0.10 (−0.50, 0.69)	−0.35 (−1.68, 0.97)
No	Yes			
PM_2.5_		1.3 (−38.2, 40.8)	−0.15 (−1.24, 0.93)	0.31 (−1.87, 2.49)
SO_4_^2−^		7.9 (−30.7, 46.4)	0.07 (−0.99, 1.12)	−0.08 (−2.22, 2.05)
NO_3_^−^		13.4 (−32.6, 59.4)	−0.04 (−1.3, 1.22)	−0.73 (−3.27, 1.81)
NH_4_^+^		11 (−33.6, 55.7)	0.10 (−1.12, 1.32)	−0.69 (−3.16, 1.77)
OM		−2.8 (−38.4, 32.8)	−0.21 (−1.18, 0.77)	0.49 (−1.47, 2.46)
BC		−3.0 (−37.0, 31.0)	−0.22 (−1.15, 0.71)	0.56 (−1.31, 2.44)
Yes	No			
PM_2.5_		21.4 (8.2, 34.5)	0.42 (0.13, 0.72)	−1.47 (−2.1, −0.84)
SO_4_^2−^		21.5 (8.8, 34.2)	0.56 (0.28, 0.85)	−1.65 (−2.26, −1.04)
NO_3_^−^		15.2 (1.1, 29.3)	0.38 (0.06, 0.70)	−1.21 (−1.88, −0.53)
NH_4_^+^		13.6 (0.1, 27.0)	0.33 (0.03, 0.64)	−1.05 (−1.69, −0.40)
OM		17.7 (6.0, 29.4)	0.29 (0.02, 0.55)	−1.12 (−1.68, −0.56)
BC		16.7 (5.6, 27.9)	0.29 (0.04, 0.54)	−1.09 (−1.62, −0.55)
No	No			
PM_2.5_		8.3 (−11.5, 28.0)	0.08 (−0.38, 0.55)	−0.88 (−1.84, 0.08)
SO_4_^2−^		5.7 (−13.0, 24.3)	0.24 (−0.20, 0.68)	−0.82 (−1.73, 0.09)
NO_3_^−^		−4.5 (−25.1, 16.1)	0.13 (−0.36, 0.61)	−0.28 (−1.29, 0.72)
NH_4_^+^		−5.4 (−25.0, 14.2)	0.08 (−0.38, 0.54)	−0.19 (−1.14, 0.76)
OM		8.9 (−8.9, 26.7)	−0.01 (−0.43, 0.41)	−0.76 (−1.63, 0.11)
BC		10.0 (−7.1, 27.0)	0.004 (−0.40, 0.41)	−0.79 (−1.62, 0.04)

PLT: platelet counts; PM_2.5_: fine particulate matter; SO_4_^2−^: sulfate; NO_3_^−^: nitrate; NH_4_^+^: ammonium; BC: black carbon; OM: organic matter; MPV: mean platelet volume; PDW: platelet distribution width. ^a^ Adjusted for age and gender (education levels, total family income, and marital status); lifestyles (smoking and drinking statuses and physical activity); and BMI; as well as personal histories of hypertension, dyslipidemia, and type 2 diabetes.

## Data Availability

The data will be made available upon request by means of a project agreement from the authors.

## Supplementary Materials

The following supporting information can be downloaded at: https://www.mdpi.com/article/10.3390/toxics12080529/s1, Table S1: Associations of the mixture of PM_2.5_ and its components with platelet parameters among non-smoking shift workers who did not use air purifiers.
