# Supplementary material for: Adverse Associations of Long-Term Exposure to PM2.5 and Its Components with Platelet Traits among Subway Shift-Workers without Air Purifier Use
Source: Toxics. 2024 Jul 23;12(8):529. doi: 10.3390/toxics12080529 (PMC11359941; doi:10.3390/toxics12080529)
Supplement: Supplementary file 1 [file toxics-12-00529-s001.zip › toxics-3089667-supplementary.pdf]

Table S1. Associations of the mixture of $PM_{2.5}$ and its components with platelet parameters among non-smoking shift-workers without air purifier use

Variables	β (95%CI) [a]
PLT	0.908(0.160, 1.657)
MPV	0.020(0.004, 0.036)
PDW	-0.0765(-0.110, -0.043)

PLT: platelet counts; $PM_{2.5}$: fine particulate matter; MPV: mean platelet volume; PDW: platelet distribution width. [a]Adjusted for age and gender, socioeconomic status (education levels, total family income, marital status), lifestyles (drinking status, physical activity), BMI as well as personal histories diseases of hypertension, dyslipidemia and type 2 diabetes.
